# Supplementary material for: Identification of the Tembusu Virus in Mosquitoes in Northern Thailand
Source: Viruses. 2023 Jun 27;15(7):1447. doi: 10.3390/v15071447 (PMC10385312; doi:10.3390/v15071447)
Supplement: Supplementary file 1 [file viruses-15-01447-s001.zip › viruses-2445721-supplementary.pdf]

Table S1. The first ten hits alignment of sample P49_TH_2019 and P73_TH_2019 obtained using NCBI BLAST® website.

P49_TH_2019

Description	Scientific Name	Max Score	Total Score	Query Cover	E value	Per. Ident	Acc. Len	Accession
Tembusu virus isolate TMUV KAN2016 NS5 gene, partial cds	Tembusu virus	385	385	99%	2e-102	97.36%	239	KX184310.1
Tembusu virus strain HNU-NX2-2019, complete genome	Tembusu virus	368	368	99%	2e-97	96.04%	10994	OP186478.1
Tembusu virus isolate YN12193 polyprotein gene, complete cds	Tembusu virus	368	368	99%	2e-97	96.04%	10278	KT607936.1
Tembusu virus strain GX2021, complete genome	Tembusu virus	363	363	99%	1e-95	95.59%	10994	OM240641.1
Tembusu virus strain SD2021, complete genome	Tembusu virus	359	359	98%	1e-94	95.56%	10995	OM240640.1
Tembusu virus strain CTLN, complete genome	Tembusu virus	359	359	98%	1e-94	95.56%	10995	MZ355579.1
Tembusu virus isolate YN2020, complete genome	Tembusu virus	353	353	98%	7e-93	95.11%	10994	OQ238827.1
Tembusu virus clone 4790 NS5 protein gene, partial cds	Tembusu virus	348	348	90%	3e-91	97.10%	232	MT125024.1
Tembusu virus strain Thai-JSL385 nonstructural protein 5 gene, partial cds	Tembusu virus	342	342	88%	1e-89	97.51%	213	KC810851.1
Tembusu virus isolate YN12115 polyprotein gene, complete cds	Tembusu virus	340	340	99%	5e-89	93.83%	10278	KT607935.1

P73_TH_2019

Description	Scientific Name	Max Score	Total Score	Query Cover	E value	Per. Ident	Acc. Len	Accession
Tembusu virus strain GX2021, complete genome	Tembusu virus	459	459	100%	2e-124	98.83%	10994	OM240641.1
Tembusu virus strain SD2021, complete genome	Tembusu virus	453	453	100%	8e-123	98.44%	10995	OM240640.1
Tembusu virus strain CTLN, complete genome	Tembusu virus	453	453	100%	8e-123	98.44%	10995	MZ355579.1
Tembusu virus isolate YN2020, complete genome	Tembusu virus	448	448	100%	4e-121	98.05%	10994	OQ238827.1
Tembusu virus isolate YN12193 polyprotein gene, complete cds	Tembusu virus	442	442	100%	2e-119	97.67%	10278	KT607936.1
Tembusu virus strain HNU-NX2-2019, complete genome	Tembusu virus	436	436	100%	8e-118	97.28%	10994	OP186478.1
Tembusu virus isolate SD14, complete genome	Tembusu virus	412	412	99%	1e-110	95.70%	11001	MH748542.1
Tembusu virus isolate TMUV KAN2016 NS5 gene, partial cds	Tembusu virus	409	409	88%	2e-109	99.12%	239	KX184310.1
Tembusu virus clone 4790 NS5 protein gene, partial cds	Tembusu virus	405	405	89%	2e-108	98.27%	232	MT125024.1
Tembusu virus isolate YN12115 polyprotein gene, complete cds	Tembusu virus	392	392	100%	2e-104	94.16%	10278	KT607935.1
